# mir-218-2 promotes glioblastomas growth, invasion and drug resistance by targeting CDC27

**DOI:** 10.18632/oncotarget.13850

**Published:** 2016-12-10

**Authors:** Zhuoying Feng, Luping Zhang, Junchen Zhou, Shuai Zhou, Li li, Xuyan Guo, Guoying Feng, Ze Ma, Wenhua Huang, Fei Huang

**Affiliations:** ^1^ Institute of Human Anatomy and Histology and Embryology, Otology & Neuroscience Center, Binzhou Medical University, Laishan District, Shandong Province, 264003,China; ^2^ Institute of Clinical Anatomy, School of Basic Medical Sciences, Southern Medical University, Guangzhou 510515, China

**Keywords:** gliomas, mir-218-2, CDC27, proliferation, invasion

## Abstract

Glioma has become a significant global health problem with substantial morbidity and mortality, underscoring the importance of elucidating its underlying molecular mechanisms. Recent studies have identified mir-218 as an anti-oncogene; however, the specific functions of mir-218-1 and mir-218-2 remain unknown, especially the latter. The objective of this study was to further investigate the role of mir-218-2 in glioma. Our results indicated that mir-218-2 is highly overexpressed in glioma. Furthermore, we showed that mir-218-2 is positively correlated with the growth, invasion, migration, and drug susceptibility (to β-lapachone) of glioma cells. *In vitro*, the overexpression of mir-218-2 promoted glioma cell proliferation, invasion, and migration. In addition, the overexpression of mir-218-2 *in vivo* was found to increase glioma tumor growth. Accordingly, the inhibition of mir-218-2 resulted in the opposite effects. Cell division cycle 27 (CDC27), the downstream target of mir-218-2, is involved in the regulation of glioma cells. Our results indicate that the overexpression of CDC27 counteracted the function of mir-218-2 in glioma cells. These novel findings provide new insight in the application of mir-218-2 as a potential glioma treatment.

## INTRODUCTION

The high incidence, rapid recurrence, and poor survival estimates of glioma makes it the most common malignant central nervous system tumor [1, 2]. Glioma is characterized by sustained proliferation, enhanced invasion and migration, and various cytogenetic and molecular abnormalities. To date, surgery [3], followed by radiotherapy [4] and chemotherapy [5], is the standard treatment regimen; however, drug susceptibility has become another intractable problem. Indeed, despite advances in cancer treatment, the prognosis for patients with glioma remains poor and <5% of patients survive for 5 years after diagnosis [3]. Moreover, the underlying molecular mechanisms have yet to be elucidated. Accordingly, there is an urgency in identifying the molecular mechanisms involved in order to identify novel targets for clinical therapies.

MicroRNAs (miRNAs) are endogenous, small non-coding RNA molecules of approximately 20–23 nucleotides. After transcription, cleavage, and biological modification with the help of a series of RNA polymerases, the single-stranded RNA is incorporated into the RNA-induced silencing complex (RISC), which is the cytoplasmic effector machine for miRNA. Upon complementary binding to the related miRNA at the untranslated 3′ region within RISC, posttranscriptional RNA silencing induces target mRNA cleavage, translational inhibition, or decay [6]. Thus, mRNA interference results in a decreased level of protein encoding thereby affecting a range of cellular processes such as proliferation, invasion, migration, and apoptosis. Several miRNAs have been found to be aberrantly expressed in glioma tumor samples compared to normal brain tissue. mir-10b [7] and mir-21 [8] are significantly increased in gliomas and the down-regulation of these miRNAs decreases the malignancy of glioma cell lines. mir-7 [9], mir-34a [9, 10], and mir-218 [11] are significantly repressed in glioma cell lines and tumor samples, modulating oncogenes and tumor suppressors through numerous direct gene interactions. Therefore, miRNAs may have great potential in the future treatment of this disease.

mir-218 is an intronic miRNA encoded by two different slit genes, mir-218-1 and mir-218-2, which are located within an intron of *SLIT2* and *SLIT3*, respectively [12]. mir-218 has been reported to be highly downregulated in some tumor samples and is thought to participate in tumor progression [13–15]. However, the expression of mir-218-1 and mir-218-2, especially for the later, as well as their functional significance in glioma remains largely unknown. mir-218-1 and mir-218-2 share the same 5p sequence as mir-218-5p, whereas they vary in 3p, which constitute mir-218-1 and mir-218-2 respectively. Here we report that mir-218-2 is highly expressed in glioma cell lines and promotes glioma growth, invasion, and migration, as well as drug susceptibility through its targeting of Cell Division Cycle 27 (CDC27).

CDC27 is one of the twelve different subunits of the anaphase-promoting complex (APC/C), a large multi-protein complex that regulates chromosome segregation and mitotic exit by ubiquitin-mediated proteolysis of cell cycle regulators [16]. Recent studies have shown that specific regions in CDC27, such as tetratricopeptide repeats (TPRs), are cross-linked to APC/C binding sites through CDC20 and Cdh1, which are coactivators involved in the APC/C-mediated ubiquitination of securin and cyclins [17]. Using bioinformatics, we have shown that the 3′-UTR of CDC27 contains two potential binding elements for mir-218-2, with a 7-nt region matching with the mir-218-2 seed region. Accordingly, we hypothesized that mir-218-2 may have a role in the regulation of CDC27 expression at the posttranscriptional level, leading to a dysfunction in APC/C and, ultimately, to abnormalities in tumor progression.

Beta-lapachone (β-lap), a novel anti-tumor agent first isolated from the lapachone tree (genus Tabebuia), has shown considerable cancer specificity by selectively increasing reactive oxygen species (ROS) and oxidative stress in breast cancer cells [18]. It has also been reported to cause cellular necrosis in pancreatic and lung cancers, as well as in hepatocellular carcinoma [19, 20]. Moreover, the reported ability of β-lap to induce ROS generation has been shown to mediate autophagic cell death in glioma U87 MG cells [21]. The results from this study indicate that overexpression of mir-218-2 results in decreased sensitivity to β-lap by decreasing ROS generation.

## RESULTS

### mir-218-2 is upregulated in both glioma cell lines and tissue specimens

**Figure 1:**
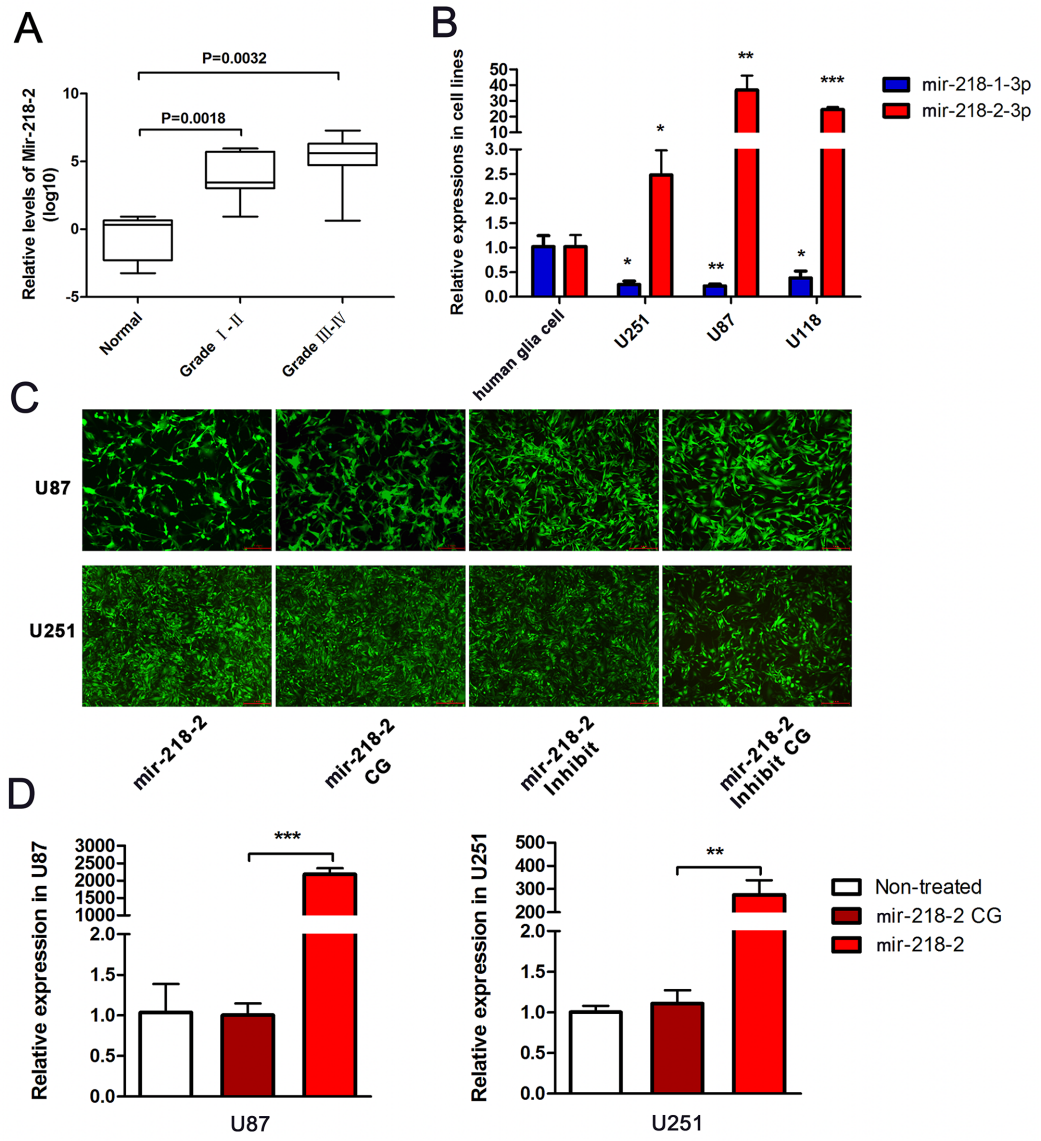
mir-218-2 is upregulated in both tissue specimens and glioma cell lines **A**. The relative expression level of mir-218-2 was measured by qRT-PCR in glioma tissue specimens compared to normal brain tissues (n ≥ 5). The expression scores are shown as box plots, with horizontal lines representing the median score; the bottom and top of the boxes represent the 25th and 75th percentiles, respectively; vertical bars represent the range of data. **B**. The relative expression level of mir-218-2 as observed in human glia cell, U251, U87, and U118 cells. *P < 0.05, **P < 0.01, ***P < 0.001. **C, D**. Lentivirus’ encoding mir-218-2 and the anti-sense to mir-218-2 were used to infect U87 and U251 cells, respectively. Green fluorescence and qRT-PCR were measured to verify the infection effect. **P < 0.01, ***P < 0.001. All data are expressed as means ± SD.

To determine the potential role of mir-218-2 in the development and progression of glioma, we first investigated the levels of mature mir-218-2 expression in five normal brain tissue samples, eight cases of grade I and II gliomas, and eleven cases of grade III-V gliomas. As is shown in Figure 1A, the expression level of mir-218-2-3p in glioma was significantly upregulated compared to the normal brain tissues and this phenomenon was particularly evident in the higher-grade glioma specimens. mir-218-2 was also overexpressed in the glioma cell lines U251, U87, and U118 compared to the human glia cell line (Figure 1B). In these cells, mir-218-1-3p was downregulated indicating that mir-218-2 may play a different role in glioma progression. In order to further investigate the potential biological function of mir-218-2 in the tumorigenesis of glioma, we infected U251 and U87 cells with a lentivirus encoding hsa-mir-218-2 or the anti-sense to has-mir-218-2 (mir-218-2 inhibited), respectively (Figure 1C and 1D).

### mir-218-2 induced glioma cell invasion, migration, and actin organization

**Figure 2:**
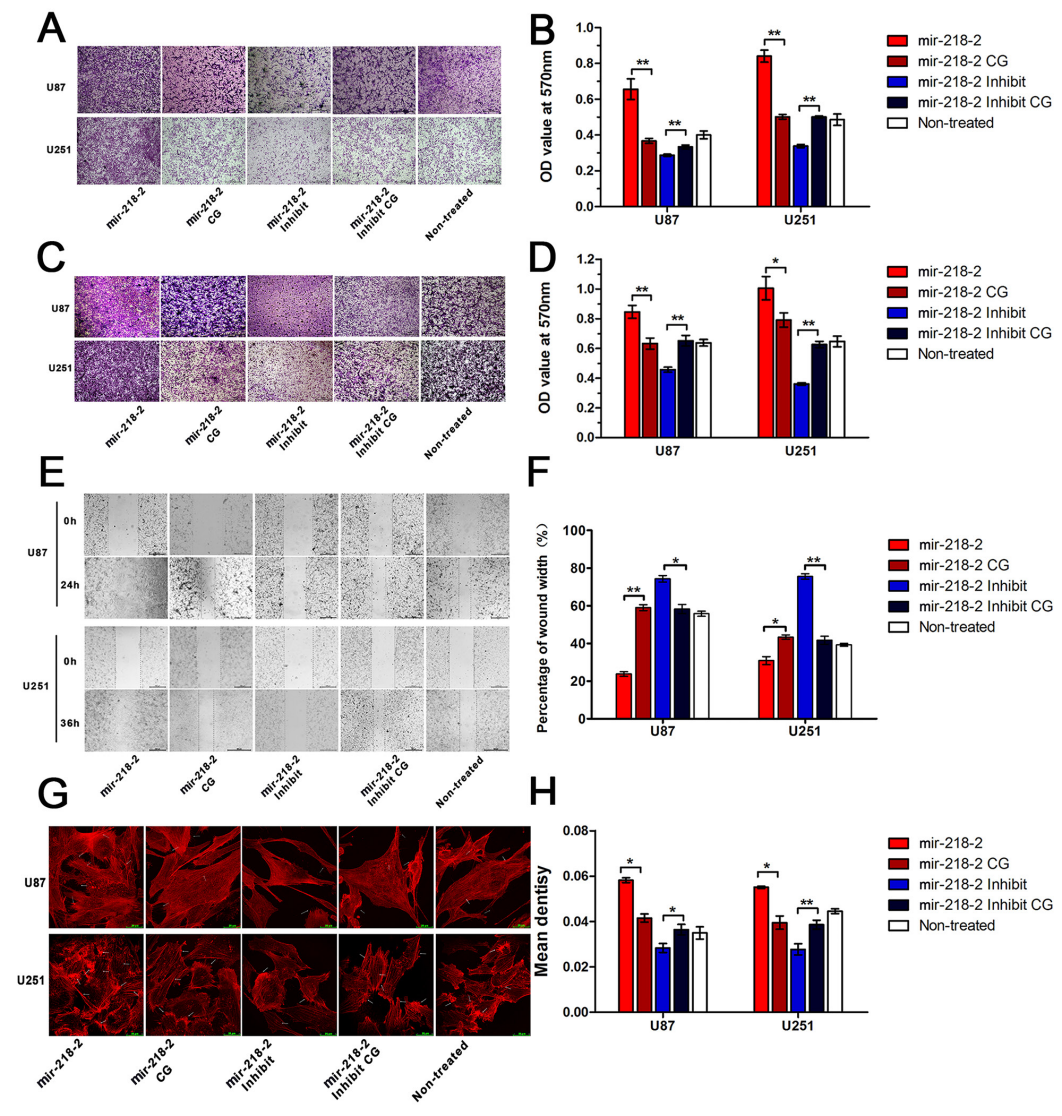
mir-218-2 induces glioma cell invasion, migration, and actin organization **A, B**. To assess the invasiveness of cells (100 × magnification) crystal violet staining was performed on the glioma cells that crossed the matrigel-coated polycarbonate membrane of the transwell chamber. An OD value of 570 nm was used as an indicator of cell number. *P < 0.05, **P < 0.01. **C, D**. Crystal violet staining of glioma cells that crossed the uncoated polycarbonate membrane of the transwell chamber to detect the migrations of cells (100 × magnification). An OD value of 570 nm was used as an indicator of cell number. *P < 0.05, **P < 0.01. **E, F**. The scratch wound assay was performed in mir-218-2 overexpressed and mir-218-2 inhibited cells. Wound closures were measured and analyzed by one-way ANOVA test. *P < 0.05, **P < 0.01. **G, H**. Staining and bar graph of F-actin by rhodamine phalloidin in the mir-218-2 overexpressed and mir-218-2 inhibited group. *P < 0.05, **P < 0.01. All data are expressed as means ± SD, n = 3.

The invasion of tumor cells through the extra cellular matrix is an important step in tumor invasion. The matrigel-coated transwell assay serves as a reconstituted basement membrane matrix and *in vitro* model system for assessing tumor cell invasiveness. As is shown in Figure 2A and 2B, the invasive capacity of U251 and U87 cells is increased upon infection with mir-218-2 and decreased when mir-218-2 expression was inhibited. No statistical differences were found among the mir-218-2 control group (mir-218-2 CG), the mir-218-2 inhibited control group (mir-218-2 inhibited CG), and the non-treated group. Next, matrigel-uncoated transwell and scratch wound assays (Figure 2C–2F) demonstrated that mir-218-2 overexpression markedly raised the invasiveness of glioma. The wound was significantly closed in cells overexpressing mir-218-2 when compared to the vector-control cells. As cytoskeleton recombination affects the adhesion and migration of cells [22], we stained the cells with rhodamine-conjugated phalloidin to visualize the changes in actin organization (Figure 2G and 2H). An increase in F-actin staining was observed in the cells overexpressing mir-218-2, with F-actin-rich membrane protrusions being clearly observable. In the mir-218-2 and CG groups, as well as in the non-treated group, stress fibers were clearly observed at the cell periphery and the cell center, and there were far fewer in the mir-218-2 inhibited group.

### mir-218-2 increases glioma proliferation and drug resistance *in vitro*

**Figure 3:**
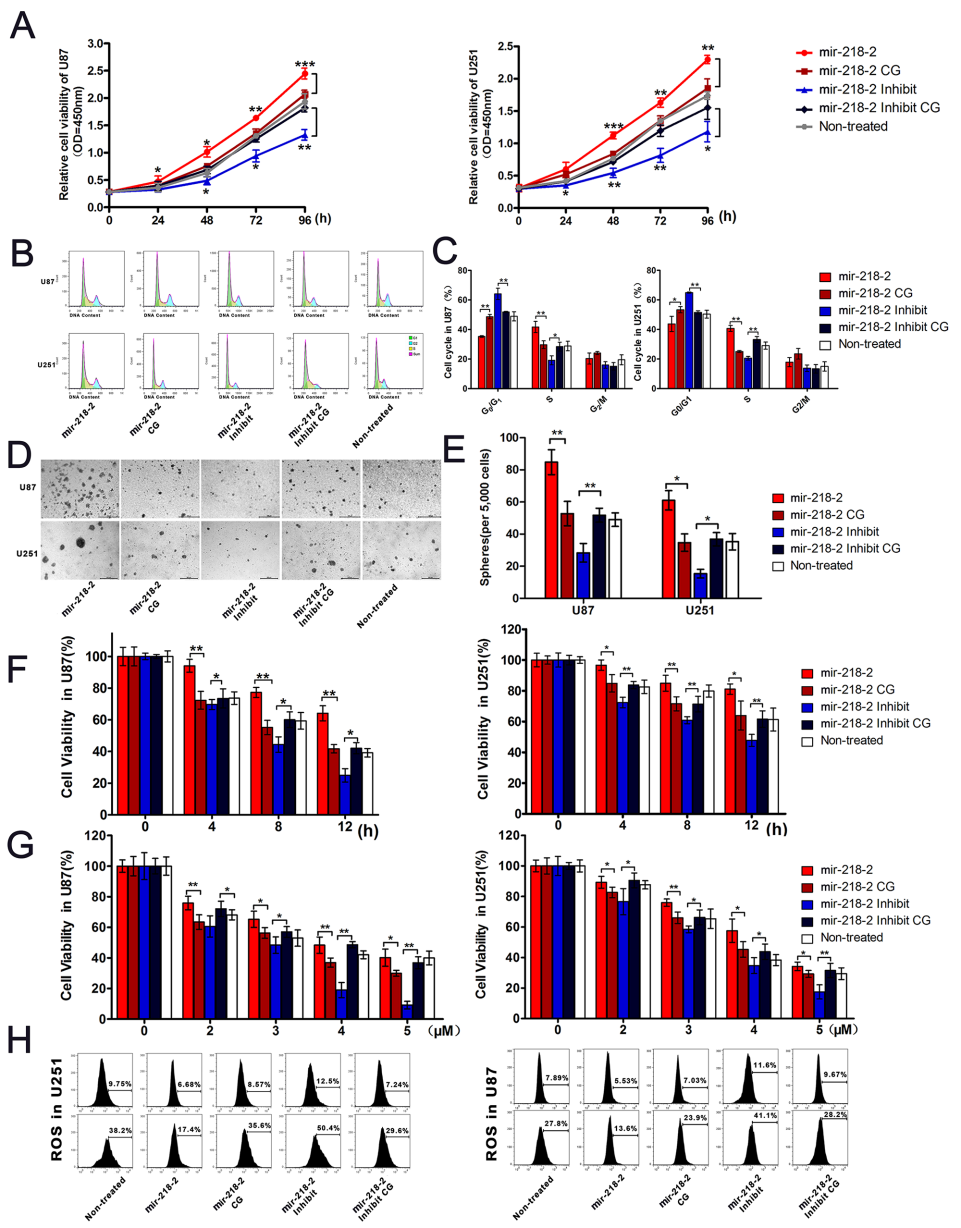
mir-218-2 increases glioma proliferation and drug resistance to β-lapchone in vitro **A**. Growth curves representing the cell viability were analyzed by the CCK8 assay. *P < 0.05, **P < 0.01, ***P < 0.001. **B, C**. Cell cycle phase distribution in mir-218-2 overexpressed and inhibited cells. The ratio is presented by the bar diagram. *P < 0.05, **P < 0.01. **D, E**. Colony formation results indicate that colony number is reduced in mir-218-2 inhibited cells. The average number of tumor clones is represented by the bar diagram. *P < 0.05, **P < 0.01. **F, G**. mir-218-2 modulated glioma cell sensitivity to β-lapchone, both in a time-dependent (0-12h) and dose-dependent (2-5 μM) manner. Cell viability was analyzed by the CCK8 assay and represented by the bar diagram. *P < 0.05, **P < 0.01. **H**. Glioma cells were treated with 4 μM β-lapachone for 1 h and coated with ROS BriteTM 670 for 1 h. The ROS BriteTM 670 density was quantified using flow cytometry. Representative data from one of three experiments yielding similar results are shown. All data are expressed as mean ± SD, n = 3.

Next, we investigated the effects of mir-218-2 on cell proliferation. As is shown in Figure 3A, the results of CCK8 assays demonstrated that the mir-218-2-inhibited glioma cells exhibited a significantly decreased rate of proliferation as compared with the CG cells. Using flow cytometry, we found that the percentage of U251 and U87 cells in S phase was significantly higher in the mir-218-2 group than in the control group (Figure 3B and 3C), and significantly lower in the mir-218-2-inhibited group. Indeed, an obvious cell cycle arrest at the G0/G1 phase was observed in the mir-218-2-inhibited group. In addition, colony formation assays demonstrated that the glioma cells overexpressing mir-218-2 generated significantly more and larger colonies than in the CG groups (Figure 3D and 3E). These data clearly demonstrate the involvement of mir-218-2 in glioma cell proliferation.

We also investigated whether mir-218-2 could modulate the anti-cancer function of β-lap, a new anti-cancer agent that has been reported to activate multiple mechanisms of cell death in cancer cells, including apoptosis [18, 19, 21]. As a result, we examined cytotoxicity using CCK8 assays. In response to β-lap, cell viability was decreased in a dose- (2–5 μM) and time- (0–12 h) dependent manner, as shown in Figure 3F and 3G. mir-218-2 overexpression significantly decreased the proportion of dead cells, whereas its knockdown enhanced apoptosis. To further verify the role of mir-218-2 in β-lap resistance, we assessed how overexpression and knockdown affected the generation of ROS with 4 μM β-lap followed by flow cytometry analysis. ROS levels, labeled using ROS Brite^TM^ 670, increased primarily in the mir-218-2-inhibited group, and less ROS were seen in the CGs and the mir-218-2 group (Figure 3H). These results suggest that mir-218-2 is involved in the regulation of β-lap chemosensitivity in glioma cells.

### CDC27 is a direct target of mir-218-2

**Figure 4:**
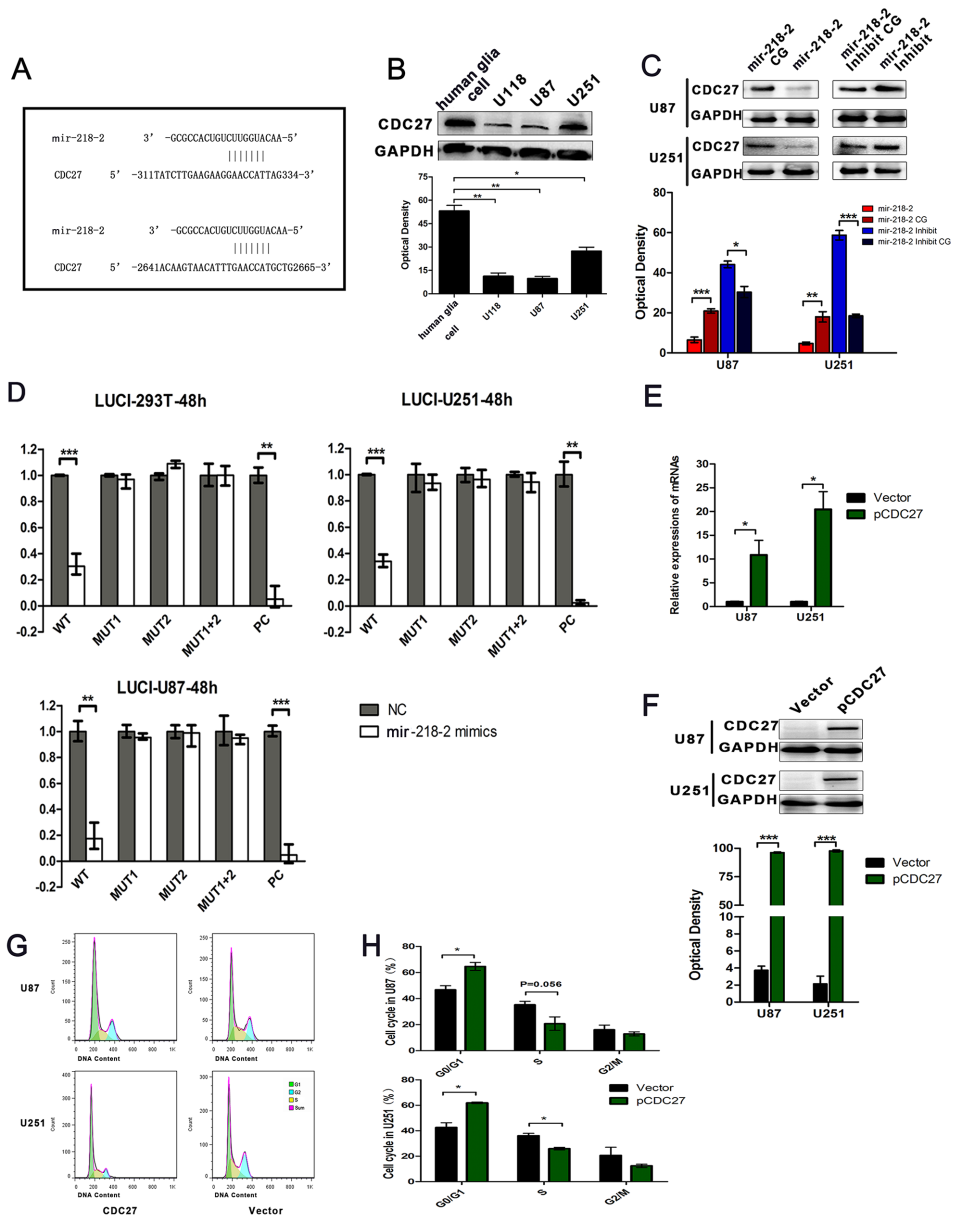
CDC27 is a direct target for mir-218-2 **A**. Schematic mir-218-2 putative target sites in the 3-UTR of CDC27. The 3-UTR of CDC27 contains two binding sites for mir-218-2. **B**. WB analysis of CDC27 protein levels in glioma cell lines compared to human glia cell line. *P < 0.05, **P < 0.01. **C**. WB analysis of CDC27 protein levels in response to mir-218-2 over- or down-expression. GAPDH was used as a loading control. *P < 0.05, **P < 0.01, ***P < 0.001. **D**. Dual-luciferase reporter assays were processed in 293T, U87, and U251 cells. *P < 0.01, ***P < 0.001. **E, F**. CDC27 was restored in mir-218-2 over-expressed cells and examined by WB and qRT-PCR, respectively. *P < 0.05, **P < 0.01, ***P < 0.001. **G, H**. Cell cycle phase distributions in CDC27 rescue and vector groups. The ratio is presented by the bar diagram. *P < 0.05. All data are expressed as means ± SD, n = 3.

Using the specific bioinformatic program MirDB (http://mirdb.org/cgi-bin/search.cgi), we got a series of targets of mir-218-2, among which we locked CDC27 for its defined function in regulating APC/C and ubiquitination in tumor. Moreover, CDC27 has got 2 binding site for Mir-218-2-3p. CDC27 was found to be a putative target of mir-218-2 with two highly conserved 3′-UTR sequences in the CDC27 3′-UTR pairing with mammalian mir-218-2 (Figure 4A). To further investigate this, we evaluated CDC27 protein levels in glioma cell lines compared to normal human glia cells. Our results indicated a decline in CDC27 protein levels in glioma cells (Figure 4B). We then verified this potential binding by performing Western blots for both the overexpressed and inhibited groups, as well as in their respective CGs. Cells infected with the mir-218-2 lentivirus had significantly decreased CDC27 expression at the protein level, whereas cells transfected with antagomir-218-2 had significantly enhanced CDC27 expression (Figure 4C). To further confirm the direct interaction between mir-218-2 and CDC27, we cloned a reporter vector in which luciferase cDNA was followed by a fragment of the 3′-UTR from the CDC27 mRNA containing the two mir-218-2 binding sites (designated as wild-type, WT). Furthermore, we synthesized additional luciferase reporters fused to the CDC27 3′-UTRs, but with mutant mir-218-2 binding sequences, called MUT1, MUT2, and MUT1+2, respectively. We then transfected these luciferase reporter vectors with either the wild-type or mutant mir-218-2 binding sequences into the 293T, U87, and U251 cell lines. We also cotransfected these cells with mir-218-2 mimic, mimic control, or positive control (PC) to measure luciferase activity. As is shown in Figure 4D, the mir-218-2 mimic (20 nM) decreased the luciferase activity of the reporter vector containing the wild-type sequences and the PC sequences. However, the mir-218-2 mimic had no significant effect on the reporter vector with the mutated binding sequences. Next, we assessed whether re-expression of CDC27 could reverse the effects of mir-218-2. To this end, we restored the expression of CDC27 in the mir-218-2 overexpressed cells with a specific plasmid (Figure 4E and 4F). As is shown in Figure 4G and 4H, the re-expression of CDC27 partially rescued mir-218-2-induced cell proliferation. Together, these data implicate CDC27 as a functionally relevant target of mir-218-2 in glioma cells.

### mir-218-2 promotes glioma progression through the CDC27/APC ubiquitin–proteasome pathway

**Figure 5:**
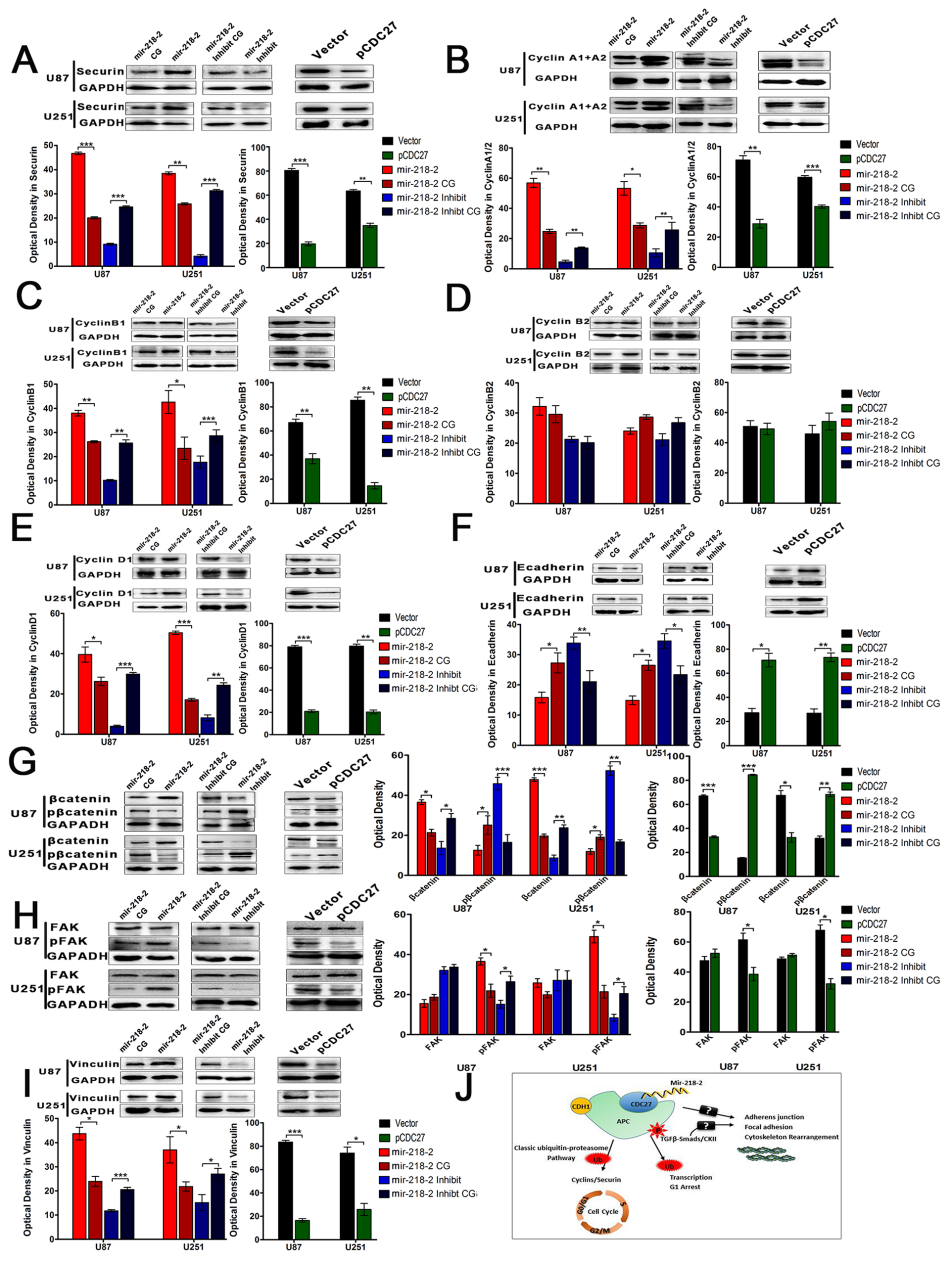
mir-218-2 promotes glioma progression through the CDC27/APC ubiquitin-proteosome pathway **A-I**. The protein expression of Securin, CyclinA1/2, CyclinB1, CyclinB2, CyclinD1, Ecadherin, βcatenin, p-βcatenin, FAK, p-FAK, and vinculin were determined by WB. Data are expressed as means ± SD, n = 3; *P < 0.05, **P < 0.01, ***P < 0.001. **J**. Schematic illustrations explain the possible targeting and signaling mechanisms by which mir-218-2 promotes glioma progression. mir-218-2 induces glioma malignancy by targeting CDC27, which leads to a decrease in the activation of the APC/C biquitin-proteosome pathway, probably downstream of the TGFβ signaling pathways. In addition, mir-218-2 may increase migration and invasion by modulating adhesion and junction proteins.

Because CDC27 is an important coactivator for APC/C-mediated ubiquitination of securin and cyclins [17], we further detected the expression of securin and several important cyclins at the protein level. As is shown in Figure 5A–5E, protein levels of securin, cyclinA1/2, cyclinB1, and cyclinD1 were increased as a consequence of CDC27 decline in the mir-218-2 group. In addition, decreasing the levels of mir-218-2 expression also decreased the levels of securin, cyclinA1/2, cyclinB1, and cyclinD1. However, the expression of cyclinB2 remained unchanged in each group. Restoration of CDC27 by transfecting the plasmid into mir-218-2 cells reversed the effects of mir-218-2 in glioma cells compared to the vector. Taken together, these data suggest that mir-218-2 modulates the APC/Cubiquitin–proteasome pathway by targeting CDC27 in glioma cells. Moreover, taking into account that adhesion and cell junction proteins, such as E-cadherin [23, 24], β-catenin [25], focal adhesion kinase (FAK)[26], and vinculin [27] play important roles in cell migration and invasion, we determined their levels of expression by performing Western blotting (Figure 5F–5I). The expression of E-cadherin, in contrast to βcatenin, was decreased in the mir-218-2 group compared to the CGs. However, phosphorylation of βcatenin, which is the deactivated form of βcatenin recognized by E3 ubiquitin ligase, was upregulated in the mir-218-2-inhibited cells. Furthermore, both vinculin and phosphorylated FAK, the activated form of FAK, were found to be upregulated in the mir-218-2 groups, whereas FAK (un-phosphorylated) maintained similar levels of expression among all groups. Taken together, our findings suggest that mir-218-2 promotes glioma progression through the CDC27/APC ubiquitin–proteasome pathway, and promotes the invasion, migration, and actin organization through the modulation of cell junction and focal adhesion proteins (Figure 5J).

### mir-218-2 accelerates glioma carcinogenesis *in vivo*

**Figure 6:**
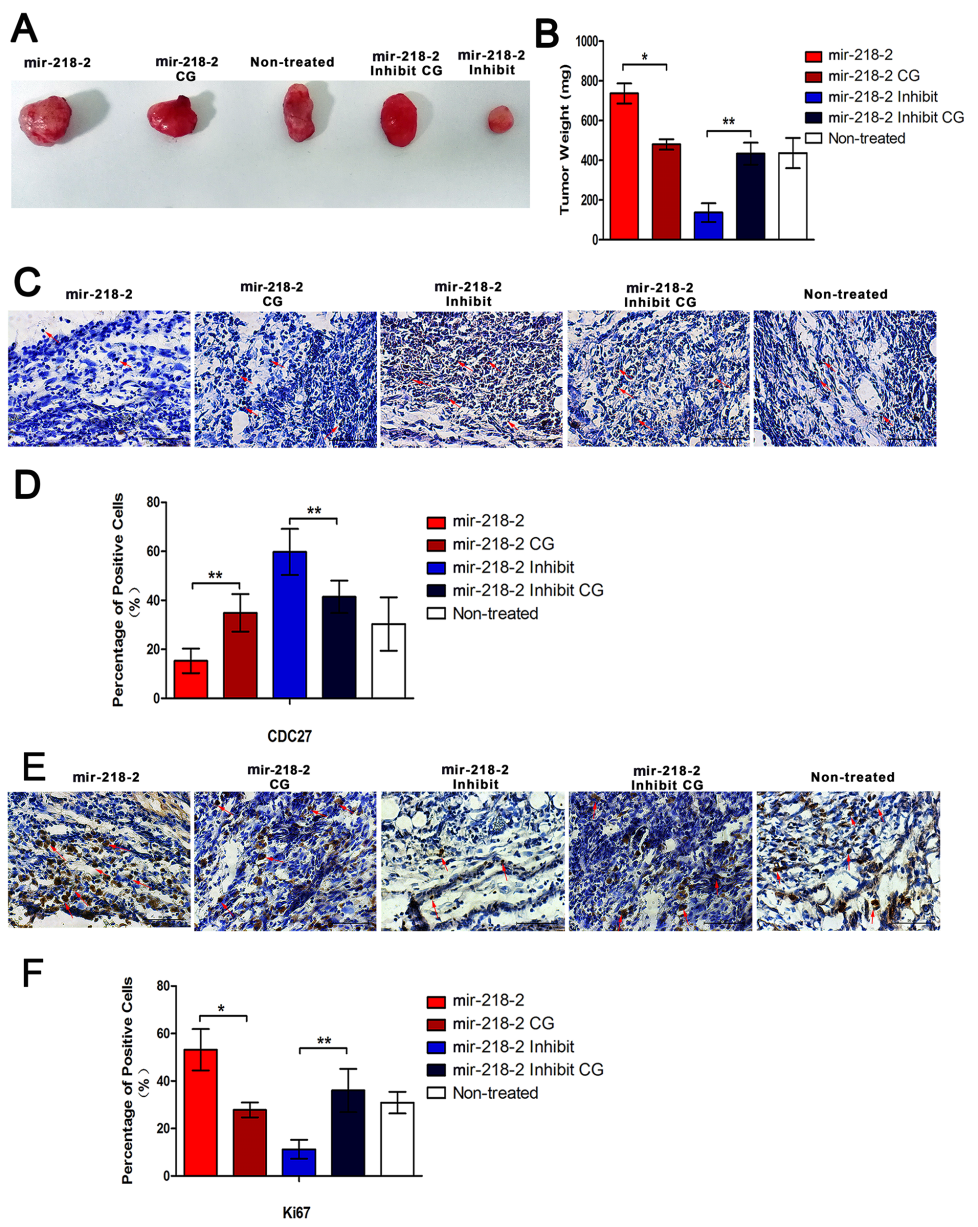
mir-218-2 accelerates glioma carcinogenesis in vivo **A, B**. Representative images of tumors and quantification of tumor weight 4 weeks after subcutaneous xenografting. Data represent means ± SD of three replicates. *P < 0.05, **P < 0.01; n = 5. **C**. Representative IHC images of CDC27 in sections at 400 × magnification. The red arrows show positive cells. **D**. Quantitation of CDC27-positive cells in sections from mir-218-2, mir-218-2 inhibited, and control mice. **E**. Representative IHC images of Ki67 in sections at 400 × magnification. The red arrows show positive cells. **F**. Quantitation of Ki67-positive cells in sections from mir-218-2, mir-218-2 inhibited, and control mice. *P < 0.05, **P < 0.01. All data are expressed as means ± SD, n= 5.

To evaluate whether mir-218-2 promotes glioma growth *in vivo*, we implanted U87 cells into the flanks of male BALB/c nude mice and weighed the tumors 30 days after injection (Figure 6A and 6B). mir-218-2-inhibited animals displayed significantly smaller tumors than CGs. Furthermore, immunohistochemistry analysis of CDC27 and a proliferation marker, Ki67, was conducted and measured in tumor xenograft tissues (Figure 6C–6F), which revealed significantly higher levels of CDC27, but lower levels of Ki67, in the mir-218-2-inhibited group. These *in vivo* observations suggest that mir-218-2 depletion decreases glioma carcinogenesis by regulating CDC27 expression.

## DISCUSSION

Despite significant improvements in the diagnosis and treatment of glioma the dismal 5-year survival rate for patients has not changed substantially, underscoring the importance for identifying and characterizing the molecular mechanisms of glioma tumorigenesis. The accumulated evidence has shown that in addition to executing various biological functions intronic sequences, such as miRNAs, also harbor functional regulatory elements [6]. Furthermore, other studies have reported that mir-218 represses the malignant transformation and progression of glioma [14] as well as other cancers, including melanoma and oral cavity squamous cell carcinomas [28, 29]. Two genes encode mature mir-218, namely mir-218-1 and mir-218-2, and are correspondingly located within introns of *SLIT2* and *SLIT3* [12]. However, the expression of mir-218-1 and mir-218-2, as well as their respective host genes, has not been well characterized for most histological types of glioma. In this study, we have uncovered several novel findings. First, we demonstrated that mir-218-2 is highly overexpressed in glioma tissue specimens and cell lines (Figure 1). Second, as shown in Figure 2, mir-218-2 was found to promote glioma invasion and migration by modulating adherent junctions, focal adhesions, and the actin cytoskeleton. Third, the up-regulation of mir-218-2 was found to induce proliferation *in vitro* (Figure 3) and *in vivo* (Figure 6). Moreover, mir-218-2 knockdown not only triggered an anti-proliferative effect in tumor growth, invasion, and migration but also enhanced chemosensitivity to β-lap, partly by inducing ROS generation. Finally, CDC27 was found to be a target gene for mir-218-2 (Figure 4), which modified the APC/C-mediated ubiquitination of cyclin proteins and securin (Figure 5). Up-regulation of mir-218-2 significantly decreased CDC27 levels in glioma cells, whereas down-regulation of this miRNA or restoring CDC27 expression induced the opposite effect. Collectively, these results may help improve our understanding of the mechanisms underlying the oncogenic effects of mir-218-2 and should impact our view of the role miRNAs have as potential therapeutic targets.

Our findings are compatible with the hypothesis that mir-218 is an important suppressor for glioma [13, 14, 30]. As was confirmed by quantitative PCR analysis, compared to the mir-218-2-3p, mir-218-1-3p was minimally expressed in glioma cell lines, indicating that mir-218-1 plays a dominant role in tumor suppression. Emerging evidence has implicated a synergistic effect between intronic miRNA and its host gene. Different from the significant inhibitory effect on tumors for *SLIT2* [31], *SLIT3* remains controversial in the progression versus suppression due to tumor species specificity. Other recent studies have validated mir-218-2 and its host gene *SLIT3* as being concomitantly downregulated in thyroid cancer. Meanwhile, the synergistic inhibitory effects of mir-218-2 and *SLIT3* have been detected in thyroid cancer cell invasion and proliferation [12]. However, another study found that slit was widely expressed in human hepatocellular carcinomas [32]. In addition, the latest research shows that *SLIT3* is expressed at significantly higher levels in higher grade gliomas than in lower grade gliomas; therefore, *SLIT3* appears promising in comparison with more traditional IHC markers currently used to diagnose glioma, particularly for the mesenchymal subtype of glioma [33]. These findings are consistent with our observations of *SLIT3* expression in glioma cells. Accordingly, we hypothesize that mir-218-2, together with its host gene *SLIT3*, contribute to glioma tumorigenesis.

CDC27, a core subunit of APC/C, interacts with mitotic checkpoint proteins and coactivates APC in the ubiquitination of securin and cyclins during mitosis [17]. Recent studies have found that securin promotes the development of aneuploidy and shortens survival for patients with human breast cancer. Importantly, CDC27 has been associated with securin expression and serve as an inverse predictor for the presence of malignant cells and shortened survival in human breast tumor cases [34]. In contrast, cyclin proteins, such as cyclin A [35], cyclin B [36] and cyclin D1 [24, 37, 38], express aberrantly in tumor species. The cyclin/CDK, cyclin A/CDK2, and cyclin D/CDK4 complexes play critical roles in cell cycle control and DNA replication. Altered activities of cyclin/CDKs are associated with the progression of various malignant human cancers. In yeast and animal cells, APC/C^Cdh1^, mediated by CDC27, targets many proteins throughout G1, including mitotic cyclins, factors that regulate spindle function, and sister chromatid cohesin proteins. In fact, lower expression CDC27 was found to be associated with poorer response to radiotherapy in squamous cell cervix carcinoma and Triple Negative Breast Cancer (TNBC) cells because of the important role of CDC27/APC in the mitotic regulatory pathway [39]. In this study, CDC27 expression, regulated by mir-218-2, was negatively correlated with the expression of most cyclins and securin, except for cyclin B2, causing stagnation in the G0/G1 phase. Meanwhile, CDC27 enhanced the chemosensitivity of glioma cells to β-lap. On the other hand, CDC27 is an inducing factor for TGF-β, which modulates APC/C activity via phosphorylation, thereby resulting in the induction of genes responsive for growth inhibition and tumor suppression [40]. The TGF-β/Smads pathways are classical pathways in both cell cycle arrest and the Epithelial-Mesenchymal Transition (EMT). It is well known that EMT is a complicated process where epithelial cells lose their cell polarity and cell-cell adhesion, thus obtaining migratory and invasive properties closely related to molecules like E-cadherin and βcatenin. However, little is known regarding how these molecules change and operate in key processes. In this study, we hypothesize that TGF-β signaling pathways may interact with E-cadherin/β-catenin and FAK/ vinculin via CDC27-APC/C, although the exact molecular mechanisms are not fully understood.

In conclusion, we found that mir-218-2 is highly overexpressed in gliomas, and that it facilitates the growth, invasion, and migration, as well as drug susceptibility of glioma cells. In addition, mir-218-2 promoted glioma carcinogenesis *in vivo*. Moreover, mir-218-2 exerted its function though the APC ubiquitin–proteasome pathway by directly targeting CDC27. Our data highlights the potential role of mir-218-2 in the prognostic evaluation and therapeutic application for glioma.

## MATERIALS AND METHODS

### Cell lines and tissue specimens

The human glia cell line (HA-1800), and glioma cell lines U251, U87, and U118 were purchased from the Type Culture Collection of the Chinese Academy of Sciences (Shanghai, China). Cell lines were maintained at 37°C in 5% CO_2_ with DMEM medium (HyClone, USA) supplemented with 10% fetal bovine serum (FBS, Gibco). A total of 19 cases of glioma (eight cases of Grade I-II and eleven cases of III-IV), which had been clinically and histologically diagnosed at the Affiliated Hospital of Binzhou Medical University between 2011 and 2015, were obtained with previous patient consent and approval from the Institutional Research Ethics Committee. Five normal brain tissue samples were obtained from individuals who suffered intracerebral hemorrhage but were confirmed to be free of any detectable pathological conditions. All tissue specimens were stored at −80°C.

### RNA extraction and real-time PCR

MiRNA expression was determined by collecting total RNA from 70% to 90% confluent cell cultures and normal or malignant human brain tissues with the MiRNeasy Mini Kit (Qiagen). The following protocol was performed as recommended in the miScript II RT Kit and miScript SYBR® Green PCR Kit (Qiagen). MiScript primers specific for mature miRNA were hsa-mir-218-1-3p and hsa-mir-218-2-3p (Qiagen). All samples were normalized to small mRNA U6 (Qiagen) and the fold change expression of miRNAs relative to U6 was determined by the 2^(-ΔΔCt)^ method. Each PCR reaction contained 12.5 μl of Fast Start Universal SYBR Green Master Mix, 2 μl of cDNA, 2.5 μl of each of 10 mM primer, and 5.5 μl ddH2O in a total volume of 25μl. Each sample was assayed in triplicate. CDC27 specific primers: (F) 5′-AGAAGTTATGTTGTGGCCTTGG-3′ and (R) 5′-AGGTACAACAGCAGCATGGTTC-3′.

### Western blotting

Cells were washed in PBS and then collected in RIPA lysis buffer (Beyotime Biotechnology, China) for protein quantification followed by addition of sample buffer and heat denaturation at 95°C for 10 min. Protein lysates were separated using 10% SDS-polyacrylamide gel electrophoresis (PAGE) gels and transferred to Immobilon-P transfer membranes (Millipore, USA). Membranes was blocked with 1% BSA supplement with 0.05% Tween-20, followed by incubation with primary antibodies overnight at 4°C. After washing with TBST, PVDF membranes were incubated with HRP-conjugated antibody for 1 h at 37°C. Immune complexes were visualized using the enhanced chemiluminescence ECL kit (Merck Millipore, USA), and detected using ChemiScope3400 Mini (Clinx Science Instruments, China). The following primary antibodies were used: anti-CDC27 antibody (Abcam, USA), anti-cyclinA antibody (Abcam, USA), anti-cyclinB1 antibody (Abcam, USA), anti-cyclin B2 antibody (Abcam, USA), anti-cyclin D1 antibody (Abcam, USA), anti-securin antibody (Abcam, USA), anti-E-Cadherin antibody (Abcam, USA), anti-beta Catenin antibody (Abcam, USA), anti-beta Catenin (phospho T41 + S45) antibody (Abcam, USA), anti-vinculin antibody (Sigma, USA), anti-FAK antibody (RD, USA), anti-FAK (phospho S732) antibody (Abcam, USA), and anti-GAPDH (1:1000, Boster Biotechnology, China).

### Lentivirus construct and plasmid transfection

Lentiviruses encoding hsa-mir-218-2 and the anti-sense to has-mir-218-2 (mir-218-2 Inhibit) with a sequence of CGCGGTGCTTGACAGAACCATG and their respective scrambled control were obtained from Genechem Inc (Shanghai, China). For re-expression of CDC27, cDNA strands corresponding to the CDC27 sequence were synthesized and cloned into the XhoI and KpnI sites of pDsRed2-Neomycin (GeneChem, Shanghai, China); a scrambled vector was used as control. For transient transfection, Lipofectamine 2000 (Invitrogen, Carlsbad, California) was used, following the manufacturer's protocol.

### Cell invasion and migration assays

The invasive and migration behaviors of indicated cells were analyzed by transwell chamber (Corning Costar Corp., Cambridge, MA, USA) assays with or without coated matrigel (BD Biosciences, Bedford, MA, USA). Cells were detached from culture plates in the absence of TrypLE™ (Gibco) and resuspended at a density of 3 × 10^5^/ml in DMEM. The lower chamber of the transwell was filled with 500 μl DMEM supplemented with 10% FBS. After 24-h incubation, the cells and gel on the upper surface were scraped using a cotton swab, whereas the cells that had invaded to the lower surface of the insert were fixed, stained with crystal violet, washed with 33% acetic acid and measured for the OD value at 570 nm, which was used as an indicator of cell number.

### Wound-healing analysis

Scratch wound assays were performed to assess the motility of indicated cells. A confluent monolayer of cells was seeded in 6-well plates. Creation of a linear scratch wound was performed using a pipette tip. After removal of cellular debris, cultures were incubated for the indicated times, and the progression of migration was observed and photographed. Image J software (National Institutes of Health, Bethesda, Maryland) was used to determine the distance between the wound edges.

### Cell proliferation assays

Cells were plated in 96-well plates in DMEM medium containing 10% FBS at a density of 5 × 104 cells/mL and incubated for 24 h. A 10 μL volume of the CCK-8 solution (Dojindo, Japan) was then added to each well and cells were cultured for another 4 h. The optical density measured at 450 nm was used as an indicator of cell viability. The plate was then read in a spectrometer at 450 nm to determine the absorbance of each well. Cell proliferation was evaluated every 24 h over a period of 5 days. Each assay was repeated three independent times in triplicate.

### Cell cycle assays

Cells were starved 12 h for synchronization followed by re-stimulation with 10% FBS for 24 h. Cells were fixed with 75% ethanol and treated according to the Cell Cycle Detection Kit (BD Biosciences, Bedford, MA, USA). Cells were then sorted by a FACS Caliber flow cytometer (Beckman, CA, USA). The cell phase distribution was analyzed with Flowjo software (Treestar Inc., USA).

### Soft agar colony formation assays

The soft agar assay was performed, to evaluate anchorage-independent growth. Cells were resuspended in 0.5 mL 0.6% low-melting-point agarose (Promega, USA) with complete culture medium, and layered on top of 0.5 mL 1.2% low melting agarose in 6-well plates (5,000 cells/well). The plates were incubated at 37°C in a humidified atmosphere of 5% CO_2_ for 2 weeks. Colonies containing at least 50 cells were counted. All experiments were repeated three times.

### Cell viability assays

Cell viability was detected with the CCK8 (Dojindo, Japan) assay. Following cultures to the log phase, cells were seeded on a 96-well plate (1 × 10^4^ cells/well). After 24 h incubation, cells were treated with various concentrations of β-lap and cultured for another 24 h. Subsequently, 10 μL of the CCK-8 solution was added to each well and measured with a microtiter plate reader at 450 nm after incubation for 3 h. For measurement of cytotoxicity in a time-dependent manner, cells were cultured with 4-μM β-lap, and cell viability was measured at 0 h, 4 h, 8 h and 12 h, respectively.

### Measurement of ROS

Intracellular accumulation of ROS was determined using the Cell Meter^TM^ Fluorimetric Intracellular Total ROS Activity Assay Kit (AAT Bioquest, USA). Cells were collected by centrifugation, treated with 4 μM β-lap for 1 h, resuspended in DMEM medium without red phenol and incubated with ROS Brite^TM^ 670 for 30 min. Fluorescence was measured with a flow cytometer (Beckman, CA, USA).

### Dual-luciferase reporter assays

293T, U87, and U251 cells were plated at 1 × 10^5^ cells per well in 24-well plates. The following day, cells were cotransfected with 250 ng of pMIR-REPORT Luciferase vector, including the 3′-UTR of CDC27 (with either wild-type or 2 mutant mir-218-2 binding sites), pmirGLO control vector, and pmirGLO with 509-inhibitor as PC, mir-218-2 mimic or mimic control at a final concentration of 20 nM using Lipofectamine 2000 (Invitrogen) according to the manufacturer's instructions. After transfection for 24 and 48 h, firefly and renilla luciferase activities were performed using the Dual-Luciferase Reporter Assay (Promega, USA). Normalized data were calculated as the quotient of renilla/firefly luciferase activities. Each experiment was repeated for at least three times in each group.

### *In vivo* tumor xenografts assays

Animal protocols were approved by the Ethical Committee of Binzhou Medicai University. Approximately (4–6) × 10^6^ U87 cells, infected or uninfected, were injected subcutaneously into the flank of male BALB/c nude mice (age 4 weeks), respectively. Each group contained five mice. Thirty days later, mice were sacrificed and tumors were excised and weighed.

### Immunohistochemistry (IHC) assays and microscopy

For F-actin staining, cultured cells were fixed in 4% methanol-free formaldehyde, washed with PBS, permeabilized in 0.1% Triton X-100, and incubated with Rhodamine phalloidin (Cytoskeleton, USA) diluted in 1% bovine serum albumin in PBS for 30 min. Micrographs were obtained using a DMI 3000 confocal microscope (Leica). Tissue sections were routinely processed and subjected to antigen retrieval. After blocking and further incubation with primary antibody at 4°C overnight, the reaction was developed using a Super Vision two-step kit (Boster, China) followed by DBA (Boster, China) staining. Harris hematoxylin (Boster, China) was used for nuclear staining at room temperature. Images were acquired with a DBI 4000B microscope (Leica) with an Imaging LAS V4.0. The following primary antibodies were used: anti-CDC27 antibody (Proteintech, USA) and anti-Ki67 antibody (Abcam, USA). Staining was measured and quantified by Image-Pro Plus (USA).

### Statistical analysis

All experiments were repeated in triplicate. Data were analyzed using the Statistical Package for Social Sciences (SPSS, version 17.0). Values shown are means ± SD. For statistical analysis, the χ2 test was used to compare categorical data and the one-way ANOVA test followed by post-hoc comparisons (Student-Newman-Keuls) were used to compare quantitative data. P values of < 0.05 were considered significant.
